# Efficacy and safety of mycophenolate mofetil in patients with immune thrombocytopenic purpura: a systematic review and meta-analysis

**DOI:** 10.1007/s10067-023-06820-4

**Published:** 2023-11-20

**Authors:** Omar Ahmed Abdelwahab, Ahmed Mechi, Shereen Gahlan, Fatima-Elzahraa Hamadein, Hallas Kadhim, Doaa Ismail, Youssef Soliman, Mohamed El‑Samahy

**Affiliations:** 1 Faculty of Medicine, Al-Azhar University, Cairo, 11884 Egypt; 2 Medical Research Group of Egypt, Cairo, Egypt; 3 Internal Medicine Department, Medicine College, University of Kufa, Najaf, Iraq; 4 Faculty of Medicine, University of Gezira, Wad Madani, Sudan; 5 College of Medicine, Al-Muthanna University, Samawah, Iraq; 6 Faculty of Medicine, Zagazig University, Zagazig, El-Sharkia, Egypt; 7 Faculty of Medicine, Assiut University, Assiut, Egypt

**Keywords:** Immune thrombocytopenic purpura, ITP, MMF, Mycophenolate mofetil, Systematic review

## Abstract

**Background:**

Immune thrombocytopenic purpura (ITP) is a challenging disease in its presentation and management as it may cause life-threatening hemorrhaging in vital organs and may resist several lines of treatment. This systematic review and meta-analysis aimed to evaluate the safety and efficacy of mycophenolate mofetil (MMF) in treating patients with ITP.

**Methods:**

We systematically searched four electronic databases (PubMed, Scopus, Web of Science, and Cochrane Central Register of Controlled Trials) from inception until 10 October 2022. We included all clinical trials, either controlled or single arm, and prospective and retrospective observational studies that evaluate the efficacy and safety of MMF in patients with ITP. We assessed the risk of bias using three tools (ROBINS-I, Cochrane ROB-2, and NIH), each for eligible study design.

**Results:**

Nine studies were included in this meta-analysis, with a total of 411 patients with ITP. We found that MMF demonstrated an overall response rate of (62.09%; 95% CI = [43.29 to 77.84]) and the complete response rate was (46.75%; 95% CI = [24.84 to 69.99]). The overall proportion of adverse events was (12%; 95% CI = [6 to 24]). After the sensitivity analysis, the overall response rate became 50%; 95% CI = [38 to 63]) and the complete response rate became (32%; 95% CI = [24 to 42]). However, MMF did not appear to affect white blood cell counts or hemoglobin levels significantly.

**Conclusion:**

This systematic review and meta-analysis demonstrate that MMF appears to be an effective and relatively safe treatment option for patients with ITP when combined with steroids and even in those who have not responded to standard therapies (steroid-resistant cases). Further research with well-designed studies is warranted to better understand the factors influencing treatment response and to refine the use of MMF in the management of ITP. An interactive version of our analysis can be accessed from here: https://databoard.shinyapps.io/mycophenolate_meta/

**Graphical abstract:**

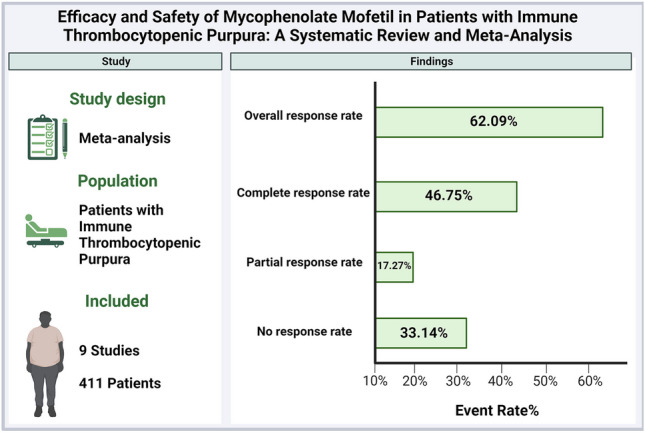

**Supplementary Information:**

The online version contains supplementary material available at 10.1007/s10067-023-06820-4.

## Background

Immune thrombocytopenic purpura (ITP) is a disorder characterized by a reduced blood platelet count, which increases the risk of hemorrhage [1]. Platelets are blood cells that serve an essential function in clotting blood [2]. They are responsible for generating clots at the area of injury in order to stop bleeding [2]. In ITP, the immune system wrongly attacks platelets, destroying them and decreasing their blood concentration [3, 4].

ITP can affect people of all ages, although children and young adults are more susceptible [5]. In children, it typically develops after a viral infection and resolves spontaneously within a few weeks to months [6, 7]. However, it can be chronic and persist for years in adults [6]. As with most autoimmune diseases, ITP is more common in females [4]; however, it is more common in male children than females [7, 8]. Depending on the severity of the disease, the ITP symptoms may vary. Some people with mild cases of ITP may have no symptoms, while others may experience bruising, petechiae (red or purple skin patches), and mucosal bleeding (bleeding from the gums, nose, or digestive tract) [9–12]. In serious instances, patients might suffer life-threatening hemorrhaging into the brain or other vital organs and also can die from a hemorrhagic shock [10, 12–14].

ITP is treated according to the disease’s severity and the presence of manifestations [11]. Treatment may not be required in mild casesand the platelet count may increase spontaneously [10]. In more severe instances, corticosteroids [15], intravenous immunoglobulin (IVIG) [16], anti-D immunoglobulin [17], and immunosuppressive agents such as mycophenolate mofetil (MMF) [18] or rituximab [19] may be administered. Some studies also suggest the use of methotrexate in ITP patients, especially in steroid-resistant cases [20, 21]. In some instances, splenectomy may also be contemplated [22].

MMF is a prodrug that the body transforms into mycophenolic acid (MPA) [23]. MPA inhibits the enzyme inosine monophosphate dehydrogenase (IMPDH) [24], which is implicated in the synthesis of guanosine nucleotides. This decreases the generation of T and B lymphocytes and the proliferation of activated lymphocytes [23, 25]. MMF is, therefore, able to suppress the immune response and has been used to treat a variety of autoimmune disorders [26], including ITP [18, 27].

This systematic review aimed to evaluate the safety and efficacy of MMF in treating patients with ITP.

## Methods

We followed the Preferred Reporting Items for Systematic Reviews and Meta-Analyses (PRISMA) statement guidelines when reporting this systematic review and meta-analysis [28]. All steps were done in strict accordance with the Cochrane Handbook of Systematic Reviews and Meta-analysis of Interventions [29]. The protocol of this study was registered on PROSPERO (CRD42023422449).

### Eligibility criteria

Studies were included in our review if they satisfied the following criteria:

- Population: studies on patients with ITP (chronic or refractory)
- Intervention: studies where the experimental (or exposed) group received MMF
- Comparator: studies where the control group did not receive MMF or received the standard therapy
- Outcome: studies reporting at least one of the following outcomes: drug response or adverse effect (headache or white blood cells (WBCs) and hemoglobin (Hb) side effects or gastrointestinal symptoms)
- Study design: we included all clinical trials, either controlled or single arm. We also included prospective and retrospective observational studies.

We excluded studies whose data were not reliable for extraction and analysis, case reports, case series, reviews, editorials, studies that were reported as a thesis, and studies that were not published in the English language.

### Information sources and search strategy

We performed a comprehensive search of four electronic databases (PubMed, Scopus, Web of Science, and Cochrane Central Register of Controlled Trials) from inception until 10 October 2022 using the following query: (“Idiopathic Thrombocytopenic Purpura” OR “Immune Thrombocytopenic Purpura” OR “Thrombocytopenic Purpura” OR “Immune Thrombocytopenia” OR “Thrombocytopenias” OR “Werlhof Disease” OR “Werlhofs Disease” OR (“Autoimmune Thrombocytopenic Purpura” OR “ITP”) AND (“Mycophenolate Mofetil” OR “Mycophenolate” OR “Mycophenolic Acid” OR “Morpholinoethyl Ester” OR “Mycophenolate Sodium” OR “Myfortic” OR “RS 61443”). Further, the references of the included studies were manually searched for any potentially eligible studies.

### Selection process

Duplicates were removed using Endnote (*Clarivate Analytics, PA, USA*), and the retrieved references were screened in two steps: the first step was to screen titles/abstracts of all identified articles independently by all authors to assess relevance to this meta-analysis, and the second step was to screen the full-text articles of the identified abstracts for final eligibility to meta-analysis.

### Data collection process and data items

Data were extracted into a uniform data extraction sheet. The extracted data included (1) a summary of the included studies (study ID, title, design, groups, country, inclusion criteria, exclusion criteria, main finding); (2) characteristics of the population of included studies (study ID, groups, number of participants, dose, age, gender (male), and follow-up); (3) risk of bias domains; and (4) outcome measures (drug response and adverse effects).

### Assessing the risk of bias in and between the individual studies

We assessed the risk of bias using three tools each for eligible study design. For the non-randomized trial, we used the risk of bias in non-randomised studies—of interventions (ROBINS-I) tool [30]. The randomized control trial was assessed by Cochrane risk of bias (ROB)-2 [31], while single-arm clinical trials were evaluated using quality assessment for before-after (pre-post) studies with no control group according to the National Institutes of Health (NIH) tool [32].

In the present study, we could not assess the existence of publication bias by Egger’s test for funnel plot asymmetry, as according to Egger and colleagues [33, 34], publication bias assessment is unreliable for < 10 pooled studies.

### Statistical analysis

Data analysis was performed using Rstudio (Version 4.2.2). To estimate the combined proportion and its 95% confidence interval (CI) for all analyzed outcomes, a random effect model was utilized. The meta prop function from the meta library within Rstudio was employed for this purpose. Examination of heterogeneity was accomplished by assessing *I*^2^ along with its corresponding *p*-value. In instances where statistically significant heterogeneity was detected, a sensitivity analysis was carried out to pinpoint the particular study responsible for introducing the heterogeneity.

For the meta-analytical approach, a random intercept logistic regression model was employed, employing maximum-likelihood estimation for tau^2. The calculation of random effects confidence intervals relied on the t-distribution, and a logit transformation was applied accordingly. Heterogeneity testing was performed using both Wald-type and likelihood-ratio tests. Notably, a continuity correction of 0.5 was implemented when dealing with studies containing zero cell frequencies, strictly for the purpose of computing individual study outcomes.

## Results

### Literature search results

**Fig. 1:**
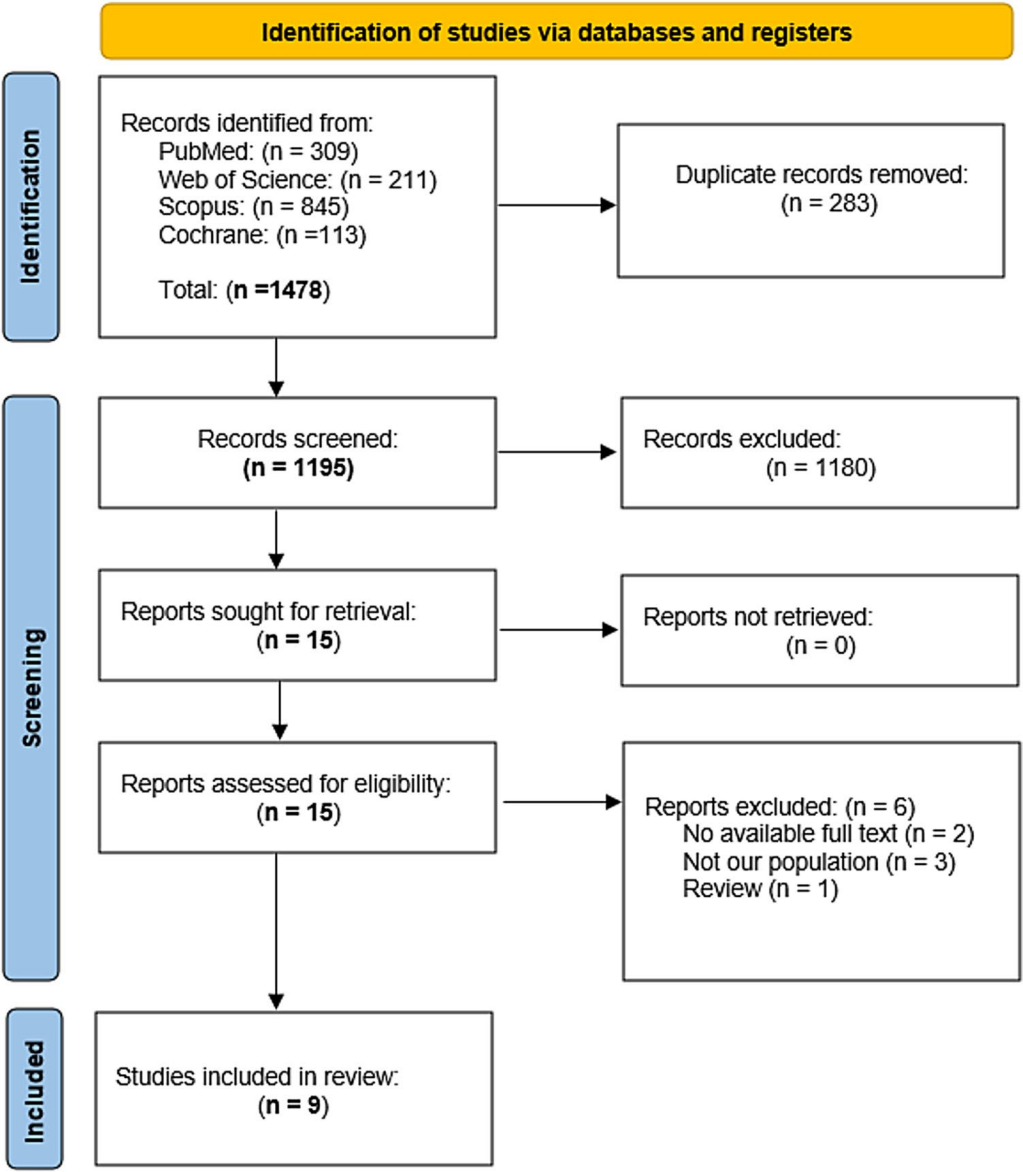
PRISMA flow diagram of studies’ screening and selection

Our literature search has shown 1229 studies. Regarding title and abstract screening, only 15 studies were included for full-text screening. Nine studies were eligible for our systematic review. No further studies were included following a manual search of the references. The PRISMA flow diagram shows all search results (Fig. 1).

### Characteristics of the included studies

Of the nine included studies, one is a randomized controlled trial, seven are single-arm clinical trials, and one is a non-randomized, controlled clinical trial. Out of the nine included studies, six included steroid-resistant ITP cases [27, 35–39] and two [18, 40] investigated MMF plus steroids compared to the steroids alone. The summary and baseline of the included studies are shown in Tables 1 and 2, respectively. The quality assessment for the single-arm clinical trials showed five studies with good quality, and two with moderate quality (Table S1). The risk of bias for randomized and non-randomized controlled trials revealed good quality (Tables S2 and S3).

**Table 1:** Summary of the studies included in this systematic review

Study ID	Design	Country	Groups	Inclusion criteria	Steroid resistant?	Main finding
Bradbury 2021 [18]	Randomized control trial	UK	MMF plus glucocorticoid	Age > 16 years. Platelet < 30 × 10^9^	NR	MMF plus glucocorticoid is associated with an effective response to immune thrombocytopenia
			glucocorticoid			
Taylor 2015 [27]	Single-arm clinical trial	UK	MMF	Patients with severe steroid-resistant ITP who started MMF treatment from 1993 to 2013	NR	MMF reduces the need for steroids even without complete remission
Provan 2006 [39]	Single-arm clinical trial	UK	MMF	Patients with refractory ITP with platelet < 20 × 10^9^	NR	MMF is effective for refractory ITP patients
Zhang 2005 [38]	Single-arm clinical trial	China	MMF	Patients with refractory ITP without viral infection, collagen vascular diseases, malignancy, or medications	20	The median dose of MMF is effective for refractory ITP patients
Arnold 2019 [37]	Single-arm clinical trial	Canada	MMF	Chronic refractory ITP with platelet < 20 × 10^9^/L for 12 months	NR	Mycophenolate, azathioprine and cyclosporine combination should be highlighted in refractory or severe ITP
Miano 2015 [41]	Single-arm clinical trial	Italy	MMF	Chronic or refractory autoimmune cytopenia plus at least one absolute or primary additional criterion for ALPS	NR	MMF and sirolimus should be highlighted in autoimmune cytopenia, either refractory or chronic, as they are safe and effective
Colovic 2010 [36]	Single-arm clinical trial	Serbia	MMF	Severe steroid-resistant ITP for 6 months	16	MMF is effective for steroid-resistant ITP patients
Hou 2003 [35]	Single-arm clinical trial	China	MMF	Severe steroid-resistant ITP not related to viral infection, collagen vascular diseases, malignancy, or medications for 6 months	21	MMF is effective for ITP patients
Xu 2019 [40]	Non-randomized clinical trial	China	MMF plus prednisone	Platelet count of less than 30 × 10^9^	NR	MMF plus prednisone is associated with an effective response to immune thrombocytopenia
			prednisone alone

**Table 2:** Baseline characteristics of the included studies

Study ID	Groups	Participants number	Dose	Age	Gender (male)	Follow-up
Bradbury 2021 [18]	MMF plus glucocorticoid	59	500 mg twice daily, then 1 g twice daily (if no adverse effect after 2 weeks)	56.9 (18–86) (mean range)	28	Median 1.3 years
	Glucocorticoid alone	61	500 mg twice daily, then 750 mg twice daily (if no adverse effect after 2 weeks)	53.1 (17–87)	37	Median 1.1 years
Taylor 2015 [27]	MMF	46	Ranged from 0.5 to 2 mg once daily (mainly 1 g)	52.5 (19–93) median and range	27	15 months (range 2–90 months)
Provan 2006 [39]	MMF	18	Initially: 0.5 g/day. After one week:1 g/day. By 3 weeks: 2 g/day. The pediatric patient’s dose was started at 250 mg/day and increased to 750 mg/day	NR	NR	2–6 months (median, 3 months)
Zhang 2005 [38]	MMF	20	Ranged from 1.5 to 2 mg once daily	median 44.3	12	NR
Miano 2015	MMF	58	NR	4.9 (0.1–16.2) Median (range)	24	3.46 (1–13) Median (range)
Arnold 2019 [37]	MMF	19	MMF 1 to 2 g/d, cyclosporine 2 mg/kg per day, and azathioprine 2 mg/kg per day	Median: 51	5	47 months (IQR, 30.0–53.0)
Colovic 2010 [36]	MMF	16	Ranged from 1.5 to 2 mg once daily	Median: 55	6	78 weeks (range 14–168 weeks)
Hou 2003 [35]	MMF	21	Ranged from 1.5 to 2 mg once daily	Median 39.6	NR	22 weeks (ranging from 12 to 56 weeks)
Xu 2019 [40]	MMF plus prednisone	48	MMF dispersible tablets twice a day, 1 g each time	NR		NR
	Prednisone alone	45	0.5 mg/kg prednisone acetate tablets twice daily	NR	59	NR

*NR* not reported, *MMF* mycophenolate mofetil

### Response to MMF

We conducted a single-arm meta-analysis to evaluate the efficacy of mycophenolate for ITP. The primary outcome measure was the combined proportion of partial and complete responses.

**Fig. 2:**
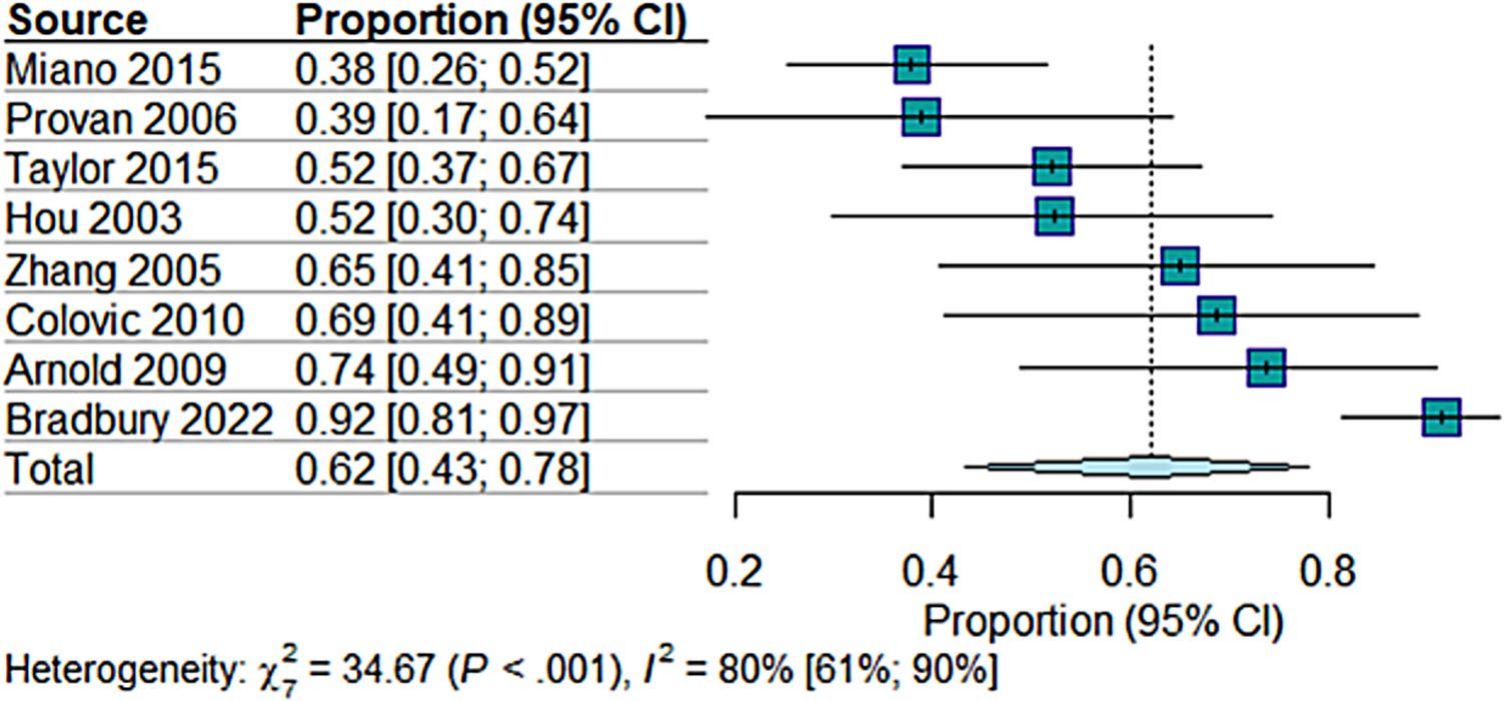
The pooled analysis of the overall response rate

For overall response, combining partial and complete response, the random effects model estimated a proportion of (62.09%; 95% CI = [43.29 to 77.84]) based on eight studies with 257 observations and 156 events. Heterogeneity was significant (*I*^2^ = 79.8%, *p* < 0.0001), suggesting considerable variation in treatment response across the studies (Fig. 2). Sensitivity analysis was done by removing the Arnold et al. 2009 [37] and Bradbury et al. 2021 [18] studies which resolved the heterogeneity (*I*^2^ = 39%, *p* = 0.15), while the effect size decreased to (50%; 95% CI = [38 to 63]) (Figure S1). The reason for these two studies introducing heterogeneity is that the intervention was MMF + glucocorticoids rather than MMF alone. This is why they had a relatively higher response rate.

**Fig. 3:**
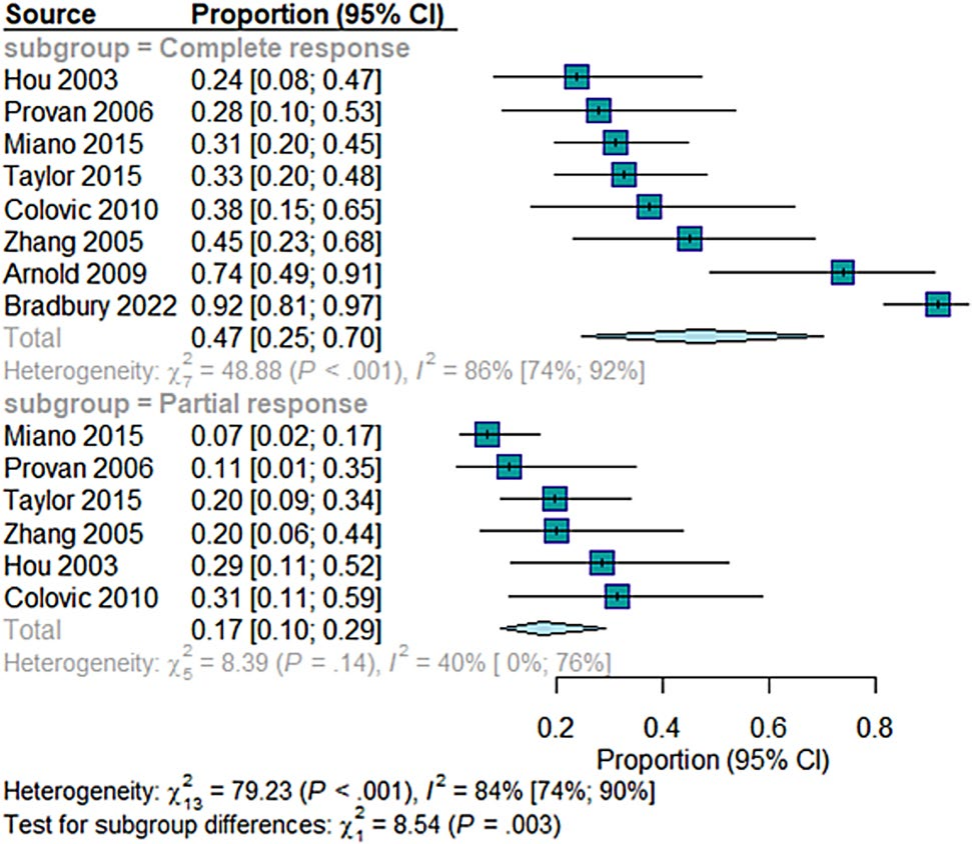
The pooled analysis of the overall response rate with subgrouping according to the response, partial or complete

Subgroup analysis based on response type revealed that the proportion of complete response was (46.75%; 95% CI = [24.84 to 69.99]) in eight studies, while the proportion of partial response was (17.27%; 95% CI = 9.53 to 29.27]) in six studies. The test for subgroup differences was statistically significant (*Q* = 8.54, df = 1, *p* = 0.0035), indicating a significant variation in response between the subgroups. There was statistically significant heterogeneity in the complete response group (*I*^2^ = 86%, *p* < 0.001) (Fig. 3). Sensitivity analysis was done by removing Arnold et al. 2009 [37] and Bradbury et al. 2021 [18] studies which resolved the heterogeneity (*I*^2^ = 0%, *p* = 0.77), while the effect size decreased to (32%; 95% CI = [24 to 42] (Figure S2).

**Fig. 4:**
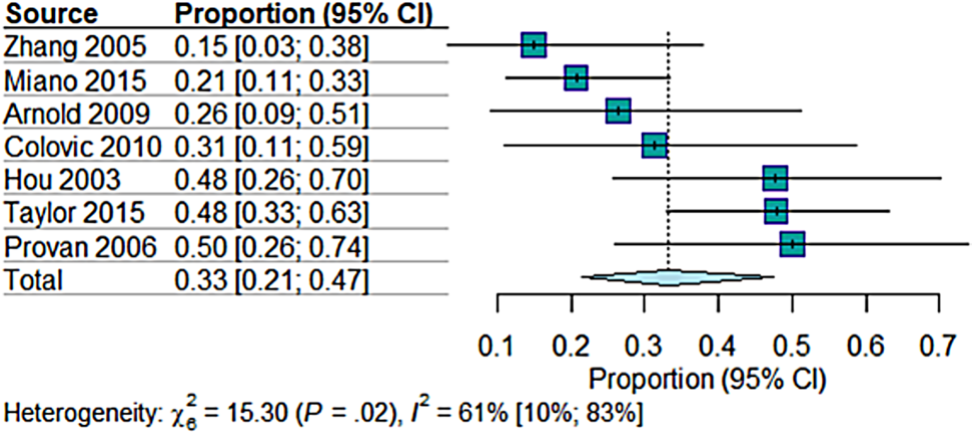
The pooled analysis of no response rate

Lastly, the proportion of non-response to mycophenolate was (33.14%; 95% CI = [21.43 to 47.39]) in seven studies with 198 observations and 66 events. The heterogeneity analysis indicated moderate heterogeneity (*I*^2^ = 61%, *p* = 0.02), implying some variation in non-response rates among the studies (Fig. 4).

### Adverse events

**Fig. 5:**
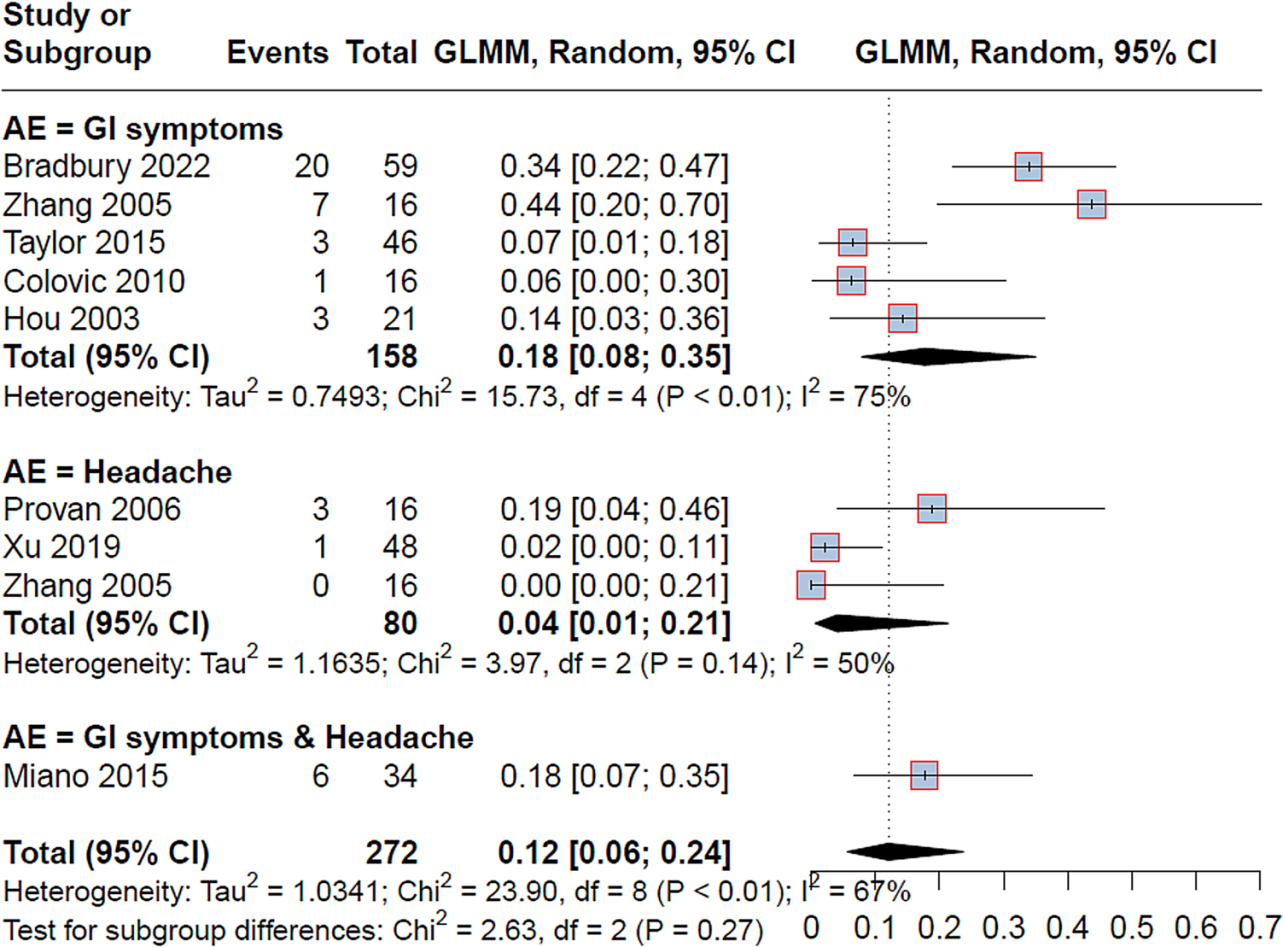
The pooled analysis of the adverse events

Eight studies reported adverse events with MMF use in a total of 272 patients with ITP. The overall proportion of adverse events was (12%; 95% CI = [6 to 24]) with significant heterogeneity (*I*^2^ = 67%, *p* < 0.01). The subgrouping according to the type of adverse events showed that the gastrointestinal adverse events (diarrhea, nausea, abdominal pain) were the most common (18%; 95% CI = [8 to 35]), then the headache (4%; 95% CI = [1 to 21]). One study (Miano et al. 2015 [41]) reported gastrointestinal adverse events and headaches as a combination with a proportion of (8%; 95% CI = [7 to 35]) (Fig. 5 and Table 3). Three studies [36, 38, 39] measured the effects of MMF on the white blood cells and hemoglobin and showed that MMF treatment did not affect any of them with a 0% in all these studies (Table 3). Additionally, the poor quality of life was reported as an adverse event of MMF in the Bradbury et al. study [18], which found that MMF-treated patients reported worse quality-of-life outcomes regarding physical function and fatigue compared to glucocorticoids-treated patients, despite the lack of difference between them in any other adverse events.

**Table 3:** Adverse events as reported by each study

Study	Gastrointestinal symptoms (diarrhea, nausea, abdominal pain)	Headache	WBCs and Hb side effects
Bradbury 2022 [18]	20 (33.9%)	NR	NR
Colovic 2010 [36]	1 (6.25%)	NR	0
Hou 2003 [35]	3 (12.28%)	NR	NR
Miano 2015 [41]	6 (15%)	NR	
Provan 2006 [39]	NR	3 (16.6%)	0
Taylor 2015 [27]	3 (6.5%)	NR	NR
Xu 2019 [40]	NR	1 (2%)	NR
Zhang 2005 [38]	7 (35%)	0	0

An interactive summary of our study findings can be accessed from here: https://databoard.shinyapps.io/mycophenolate_meta/

## Discussion

### Significance of the study

This systematic review and meta-analysis aimed to evaluate the efficacy of MMF in treating patients with ITP, a challenging autoimmune disorder. Understanding the role of MMF in ITP management is crucial as it can potentially provide an alternative or adjunctive therapy for patients who are unresponsive to standard treatments.

### Summary of findings

Our study included nine studies with a total of 411 patients with ITP. In our analysis, we found that MMF demonstrated an overall response rate (combining partial and complete responses) of 62.09%. This suggests that MMF may be effective in inducing a response in a significant proportion of ITP patients. Subgroup analysis revealed that the proportion of complete response was 46.75%, while the proportion of partial response was 17.27%, indicating a variation in treatment response, with the complete response being predominant. Furthermore, the proportion of non-response to mycophenolate was 33.14%. Regarding adverse events, MMF use was associated with an overall proportion of 12% adverse events, with gastrointestinal symptoms being the most common, followed by headaches. Notably, MMF did not appear to significantly affect white blood cell counts or hemoglobin levels.

Of the included studies, two were controlled studies [18, 40] and compared MMF and steroids in treating patients with ITP. Xu et al. [40] compared the MMF plus prednisone with prednisone alone and found that the combination significantly increased the platelet levels when compared to the prednisone alone (*p* < 0.05). Additionally, the time to reach the normal platelets level was shorter in the combination group. Bradbury et al. [18] also investigated the use of MMF in combination with glucocorticoids as a first-line therapy in the management of ITP compared with glucocorticoids alone, with a follow-up of 2 years. The authors found that the MMF-glucocorticoids group had fewer treatment failures than glucocorticoids alone. Additionally, the combination group had a greater response than the glucocorticoids alone group. However, the patients in the combination group had worsened quality-of-life outcomes in terms of physical function and tiredness.

### Explanation of the finding and potential mechanisms of MMF in ITP

The observed overall response rate of 62.09% with significant heterogeneity indicates that MMF has a considerable impact on ITP management, but treatment response varies among individual patients. To understand the potential mechanisms by which MMF exerts its effects in ITP, we need to consider its pharmacological properties and its known immunomodulatory actions.

MMF is an immunosuppressive medication that inhibits IMPDH [23], an enzyme that is required for the de novo synthesis of guanosine nucleotides, specifically guanosine monophosphate (GMP) and guanosine diphosphate (GDP) [23, 25]. These nucleotides are essential for deoxyribonucleic acid (DNA) synthesis and cell proliferation, especially in rapidly dividing cells such as lymphocytes [42].

#### Suppression of B and T lymphocytes

Lymphocytes play an important part in the development of ITP [43], and MMF predominantly targets them [23, 44]. T and B lymphocytes are involved in the destruction of platelets in ITP, either directly or indirectly, by developing autoantibodies against platelet surface antigens [12]. MMF suppresses lymphocyte proliferation by lowering the availability of GMP and GDP, hence restricting DNA synthesis and cell division [23, 25]. This activity reduces the number of autoreactive lymphocytes, resulting in less platelet destruction and possibly higher platelet counts [44].

#### Autoantibody production modulation

Autoantibodies that target platelet surface antigens are produced by B lymphocytes in ITP patients, resulting in platelet destruction via the reticuloendothelial system [45–48]. MMF may inhibit the generation of autoantibodies against platelets by decreasing B cell proliferation and antibody synthesis, contributing to the stabilization or enhancement of platelet levels [49].

#### Immunomodulatory actions

MMF’s immunomodulatory activities go beyond the inhibition of lymphocytes [43, 44]. It also has an impact on other immune cells, such as dendritic cells and macrophages [50, 51], which play important roles in immune response and inflammation regulation [52]. MMF may help reduce the inflammatory component of ITP by slowing the activity of these cells, lowering platelet destruction, and increasing platelet survival.

#### Impact on regulatory T cells (Tregs)

Tregs are a type of T cell renowned for their immunosuppressive properties, including the suppression of autoreactive T cells [53, 54]. MMF therapy has been linked to an increase in Treg frequency, which could improve their suppressive action [55]. MMF may enhance immunological tolerance and alleviate the autoimmune response that causes ITP by fostering a more favorable Treg-to-effector T cell balance.

#### Anti-inflammatory effects

MMF has anti-inflammatory properties in addition to its direct immunosuppressive effects [56–58]. Pro-inflammatory cytokines and chemokines play a role in platelet destruction and decreased platelet synthesis in ITP [59–61]. The anti-inflammatory effects of MMF may aid in the reduction of inflammatory mediators, resulting in a more balanced immunological milieu and enhanced platelet homeostasis [56–58, 62].

#### Platelet-associated autoantigen reduction

Platelets may carry autoantigens on their surface in some cases of ITP, which are recognized by autoreactive T lymphocytes and cause their death [11, 63, 64]. MMF’s effect on B cells and autoantibody synthesis may indirectly diminish platelet coating with autoantigens, reducing identification and destruction by immune cells [49].

It is crucial to highlight that the precise processes by which MMF operates in ITP are not fully understood, and it is likely that a combination of the aforementioned mechanisms, as well as patient-specific factors, contributes to treatment response. Furthermore, the study’s great variation in response rates highlights the need for additional research to uncover predictive markers for MMF responsiveness in ITP.

### First-line versus steroid resistant

Steroid-resistant ITP represents a complex subset of patients within the broader ITP population. These people do not have a satisfactory increase in platelet counts in response to normal corticosteroid medication [65], which is commonly used as first-line therapy for ITP [15]. Steroid-resistant ITP presents distinct challenges, necessitating a specialized approach to address the underlying immunological dysregulation that leads to recurrent platelet destruction [66–68]. When corticosteroids fail to provide the intended response, a variety of other therapeutic options are tried. One strategy involves studying several immunosuppressive drugs, such as MMF, which was the focus of this study [49, 67, 68]. MMF’s methods of action, which were described, make it a possible candidate for treating steroid-resistant cases. Out of the nine included studies, six included steroid-resistant ITP cases [27, 35–39], and two [18, 40] investigated MMF plus steroids compared to the steroids alone. When compared to the steroids, the combination of MMF plus steroids showed significantly increased platelet levels [40], a shorter time to reach the normal platelet level [40], fewer treatment failures, and a greater response rate [18].

A previous systematic review by Bylsma et al. reported the potential treatments used for ITP in a second-line setting; MMF was one of those potential drugs [69]. It was also reported as second-line therapy in the International Consensus Report [70] and the American Society of Hematology guidelines on managing primary ITP [71], but as medical therapy with less robust evidence. However, one of the additions of our study is highlighting MMF as a potential second-line therapy with more substantial evidence and even a potential first-line therapy combined with glucocorticoids.

### Recommendations for future research and clinical practice

In order to better study the efficacy and safety of MMF in treating ITP, future research should focus on conducting well-designed randomized controlled trials with bigger sample sizes. These studies should also look into potential predictors of treatment response in order to identify patient subgroups most likely to benefit from MMF therapy. Long-term follow-up studies are also required to determine how long the response lasts and to track any late adverse events that may arise. Additionally, the future research should compare MMF with other agents that are highlighted as a second-line therapy. Moreover, there is a critical need to define the specific patient characteristics or biomarkers that can reliably predict the response to MMF or other therapeutic agents. By elucidating the predictive factors associated with treatment response, such as disease phenotype, immunological parameters, or genetic markers, future research can facilitate the implementation of personalized treatment approaches, thereby maximizing the therapeutic benefits and minimizing the risks for patients with ITP.

In clinical practice, the findings of this study can guide clinicians in considering MMF as a treatment option for ITP patients, especially in steroid-resistant cases, to avoid splenectomy or even to do a safe splenectomy. Furthermore, the integration of these findings into clinical practice has the potential to inform the development of evidence-based treatment guidelines and protocols, empowering healthcare providers to make informed, data-driven decisions and optimize the standard of care for patients with ITP.

Finally, MMF is highlighted as a low-cost [36], which ensures accessibility and affordability for patients, contributing to a more efficient healthcare approach, especially a low and middle income nations.

### Strength points and limitations

To the best of our knowledge, this is the first systematic review and meta-analysis to evaluate the efficacy of MMF in treating patients with ITP. The inclusion of various study designs and the large sample size provide a comprehensive evaluation of MMF in ITP management. However, some limitations should be acknowledged. First, the included studies might have had inherent biases that could influence the overall results. Although efforts were made to assess the risk of bias using appropriate tools, the possibility of residual confounding cannot be entirely ruled out. Second, the significant heterogeneity observed in treatment response highlights the need for caution when interpreting the overall response rate. Third, the number of included studies may have been limited, with a sample size that may not be considered large sufficiently, which could affect the generalizability of the findings.

## Conclusion

This systematic review and meta-analysis demonstrate that MMF appears to be an effective and relatively safe treatment option for patients with ITP when combined with steroids and even in those who have not responded to standard therapies (steroid-resistant cases). The findings support the potential use of MMF as an alternative or adjunctive treatment for ITP, but careful patient selection and monitoring for adverse events are essential for optimizing treatment outcomes. Further research with well-designed studies is warranted to better understand the factors influencing treatment response and to refine the use of MMF in the management of ITP.

### Supplementary Information

Below is the link to the electronic supplementary material.

Supplementary file1 (DOCX 46 KB)

## Data Availability

The datasets used and/or analyzed during the current study are available as MS Excel (.xlsx) and RevMan files (.rm5) from the corresponding author on reasonable request.

## Abbreviations

*ITP*: Immune thrombocytopenic purpura
*MMF*: Mycophenolate mofetil
*ROBINS-I*: Risk of bias in non-randomised studies—of interventions
*ROB*: Risk of bias
*NIH*: National Institutes of Health
*IVIG*: Immunoglobulin
*MPA*: Mycophenolic acid
*IMPDH*: Inosine monophosphate dehydrogenase
*PRISMA*: Preferred reporting items for systematic reviews and meta-analyses
*WBCs*: White blood cells
*Hb*: Hemoglobin
*CI*: Confidence interval
*GMP*: Guanosine monophosphate
*GDP*: Guanosine diphosphate
*DNA*: Deoxyribonucleic acid
*Tregs*: Regulatory T cells
