# Supplementary material for: Efficacy and safety of mycophenolate mofetil in patients with immune thrombocytopenic purpura: a systematic review and meta-analysis
Source: Clin Rheumatol. 2023 Nov 20;43(2):621–32. doi: 10.1007/s10067-023-06820-4 (PMC10834632; doi:10.1007/s10067-023-06820-4)
Supplement: Supplementary file 1 — Supplementary file1 (DOCX 46 KB) [file 10067_2023_6820_MOESM1_ESM.docx]

# **Table S1: Risk of bias and quality assessment for single-arm clinical trials**

| **Study ID** | **Quality assessment for before-after (Pre-Post) Studies with no control group according to NIH tool** | | | | | | | | | | | | |
| --- | --- | --- | --- | --- | --- | --- | --- | --- | --- | --- | --- | --- | --- |
|  | **Q1** | **Q2** | **Q3** | **Q4** | **Q5** | **Q6** | **Q7** | **Q8** | **Q9** | **Q10** | **Q11** | **Q12** | **Overall quality** |
| **Colovic 2010** | Y | Y | N | Y | NR | Y | Y | NR | Y | Y | NR | Y | Moderate |
| **Hou 2003** | Y | Y | N | Y | NR | Y | Y | NR | Y | Y | NR | Y | Moderate |
| **Miano 2015** | Y | Y | Y | Y | NR | Y | Y | NR | Y | Y | Y | Y | Good |
| **Provan 2005** | Y | Y | Y | Y | NR | Y | Y | NR | Y | N | Y | Y | Good |
| **Taylor 2015** | Y | Y | Y | Y | NR | Y | Y | NR | Y | N | Y | Y | Good |
| **Zhang 2005** | Y | Y | N | Y | NR | Y | Y | NR | Y | Y | Y | Y | Good |
| **Arnold 2009** | Y | Y | N | Y | NR | Y | Y | NR | Y | N | NR | Y | Moderate |

**NIH tool for pre and post-studies**: Q1: Objective clearly stated; Q2: eligibility criteria described; Q3: representative patient population; Q4: all eligible participants enrolled in study; Q5: sufficient sample size; Q6: intervention described; Q7: outcome measures specified; Q8: outcome assessors blinded; Q9: loss to follow-up less than 20%; Q10: statistical analysis of outcome; Q11: interrupted time-series design; Q12: individual data used for group-level effects.

# **Table S2: Risk of bias and quality assessment for RCT**

| Study ID | D1 | D2 | D3 | D4 | D5 | Overall bias |
| --- | --- | --- | --- | --- | --- | --- |
| Bradbury 2022 | Low risk | Low risk | Low risk | Low risk | Low risk | Low risk |

**ROB2 tool**: bias due to/ in: D1: randomization process; D2: deviations from intended interventions; D3: measurement of the outcome; D4: missing outcome data; D5: selection of the reported result.

**Table S3: Risk of bias and quality assessment for non-randomized studies of interventions:**

| **Study ID** | **D1** | **D2** | **D3** | **D4** | **D5** | **D6** | **D7** | **Overall bias** |
| --- | --- | --- | --- | --- | --- | --- | --- | --- |
| **Xu 2019** | Low | Low | Low | Low | Low | Low | Low | Low |

**ROBINS1 tool**: bias due to/ in: D1: confounding; D2: selection of participants into the study; D3: classification of interventions; D4: deviations from intended interventions; D5: missing data; D6: measurement of outcomes; D7: selection of the reported result.


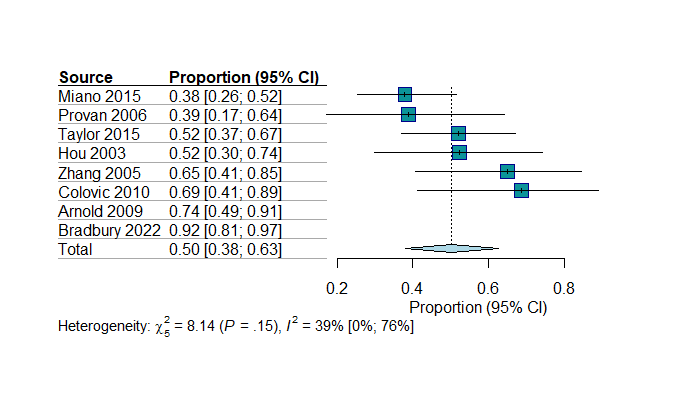


**Figure S1.** Sensitivity analysis for pooled overall response.


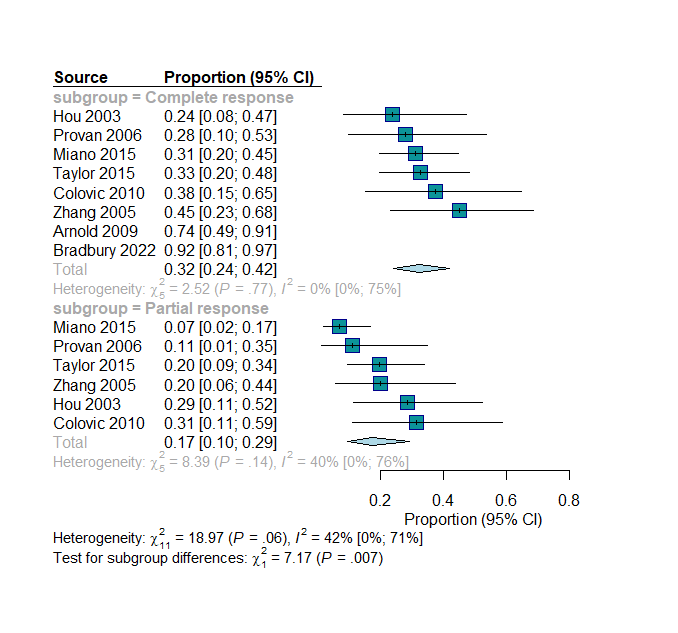


**Figure S2.** Sensitivity analysis for subgrouping of response rate
